# ST-Segment Elevation Myocardial Infarction and Coronary Artery Pseudoaneurysm

**DOI:** 10.1016/j.jaccas.2025.104883

**Published:** 2025-09-03

**Authors:** Esra Polat, Murat Abdulhamit Erçişli, Mehmet Ali Mendi, Sedat Sakallı, Engin Dondurmacı, Mehmet Onay

**Affiliations:** a Department of Cardiology, Gaziantep City Hospital, Gaziantep, Turkey; b Department of Cardiovascular Surgery, Gaziantep City Hospital, Gaziantep, Turkey; c Department of Radiology, Gaziantep City Hospital, Gaziantep, Turkey

**Keywords:** coronary artery bypass, coronary vessel anomaly, myocardial infarction

## Abstract

**Background:**

Coronary artery pseudoaneurysms (CAP) are rare, especially without any history of coronary angioplasty and coronary bypass graft. The symptoms range from asymptomatic to cardiogenic shock. Because of its rarity and variable symptoms, patients with CAP should be treated with an individualized approach.

**Case Summary:**

A 27-year-old man was admitted to the emergency department with chest pain. Electrocardiography revealed ST-segment elevation myocardial infarction in leads 2, 3, and AVF. Transthoracic echocardiography revealed a 5.5 × 4.6 cm mass filling the right atrium. Coronary angiography performed in a patient with persistent angina showed increased opacity in the right coronary artery after opaque injection in the region corresponding to the right atrium after the crux. The patient with persistent angina and hypotensive status underwent emergency surgery, and an intraoperative CAP was identified.

**Discussion:**

Coronary artery pseudoaneurysm (CAP) may be iatrogenic, due to musculoskeletal pathologies, genetic, and symptomatic depending on the location. Our patient showed that CAP can present with ST-segment elevation myocardial infarction without any etiology.

**Take-Home Message:**

A CAP is rare, but it can occur non-iatrogenically.

## Graphical Abstract

**Figure:**
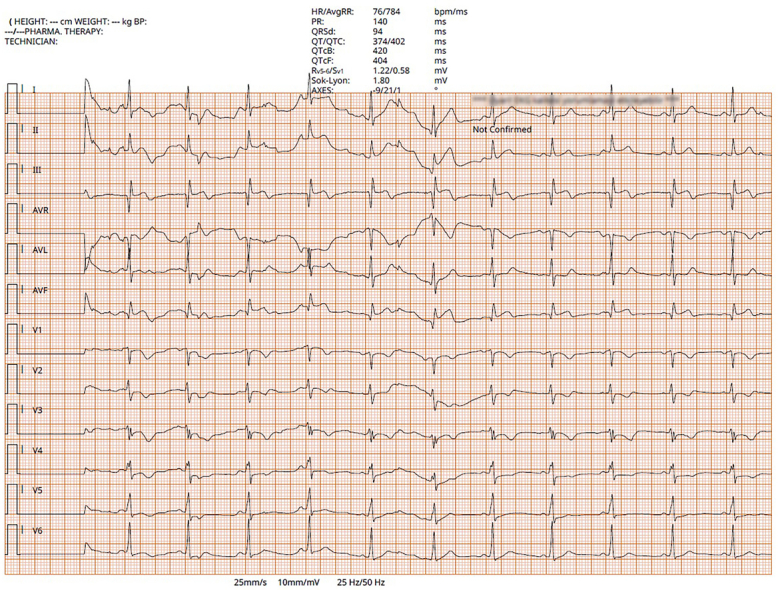

## History of Presentation

**Figure 1:**
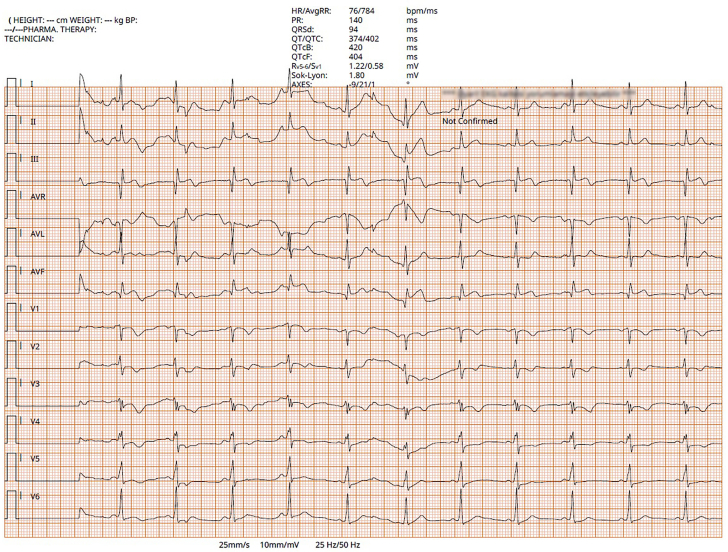
Patient's Electrocardiography

A 27-year-old man presented to the emergency department with chest pain that had been present for the last 1 week and increased especially in the last 4 hours. Electrocardiography showed ST-segment elevation in leads 2, 3, and AVF (Figure 1).

## Past Medical History

The patient had deep vein thrombosis in the left lower extremity 2 years ago after a long trip. Pulmonary computed tomographic angiography (CTA) performed at that time showed no pulmonary embolism. Transthoracic echocardiography (TTE) performed at that time showed an ejection fraction of 60%, normal right heart size, and no abnormal pathologic findings in the right heart chambers. The patient was thoroughly evaluated by the hematology and rheumatology departments to determine the duration of anticoagulant use, and no hematologic or rheumatologic disease was detected.

## Investigations

**Figure 2:**
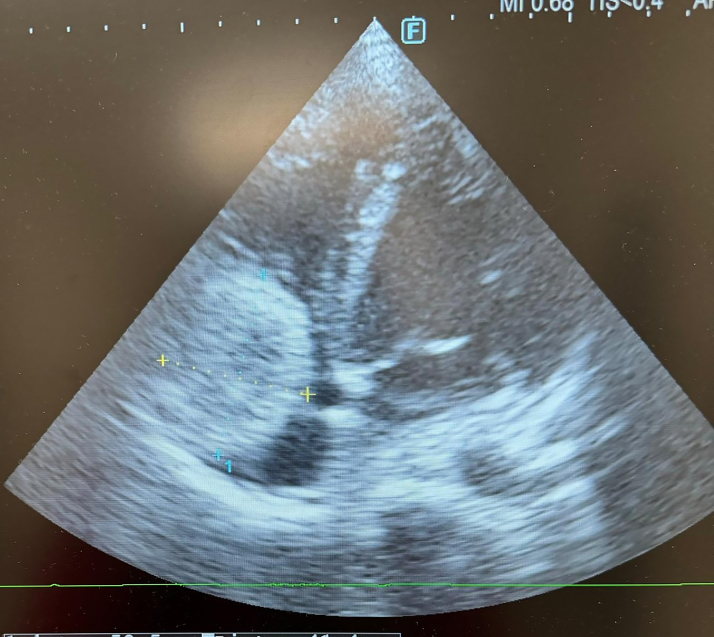
Echocardiographic Image of a Mass in the Right Atrium

**Figure 3:**
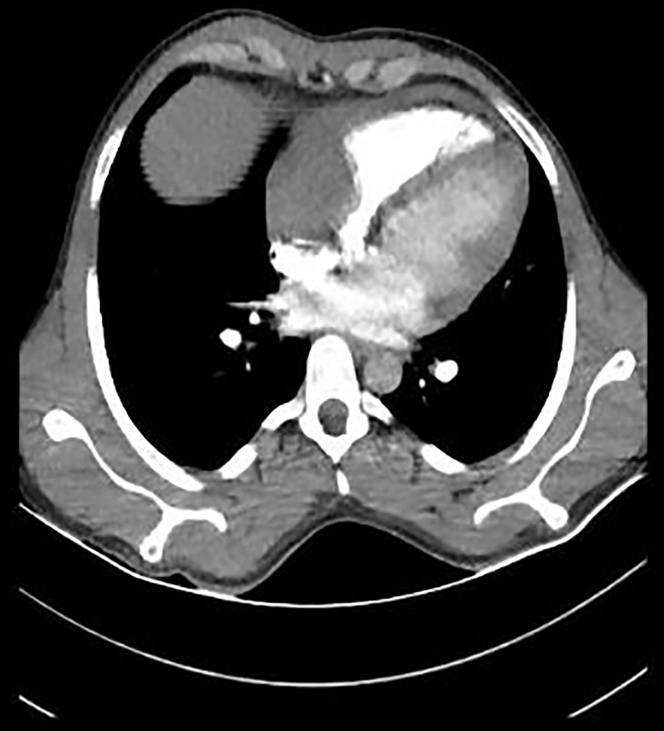
Pulmonary Computed Tomographic Angiography Shows a Space-Occupying Lesion That Largely Fills the Right Atrium and Partially Compresses the Right Ventricle

**Figure 4:**
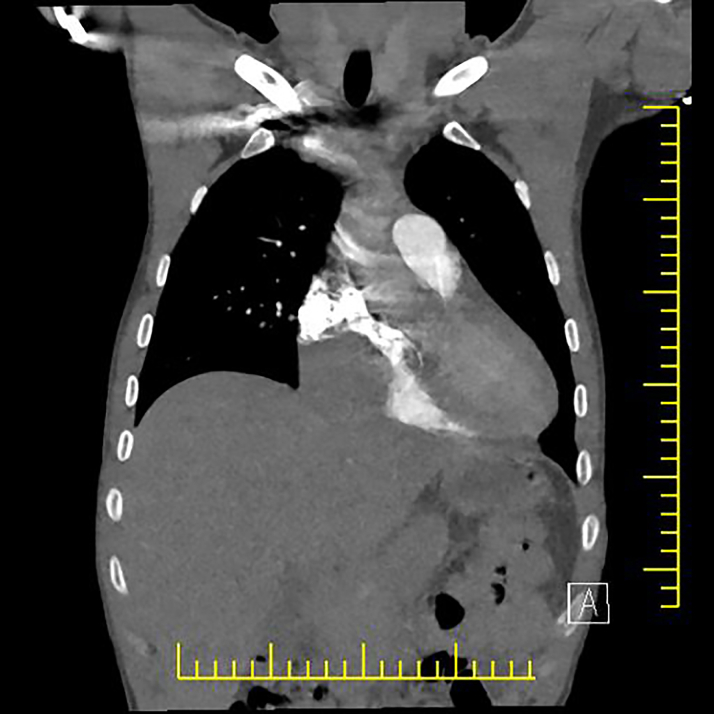
Mass Compressing the Right Ventricle on Pulmonary Computed Tomographic Angiography

In the patient with ST-segment elevation myocardial infarction (STEMI) in the inferior leads on electrocardiography, TTE revealed a 5.5 × 4.6 cm mass filling the right atrium (RA) (Figure 2; Video 1). Pulmonary CTA showed a space-occupying lesion that largely filled the RA and partially compressed the right ventricle (RV) (Figures 3 and 4).

## Management

Coronary angiography performed in the patient with persistent chest pain showed increased opacity in the right coronary artery (RCA) after opaque injection in the area corresponding to the RA after the crux (Video 2). Emergency surgery was recommended for the patient with persistent chest pain.

**Figure 5:**
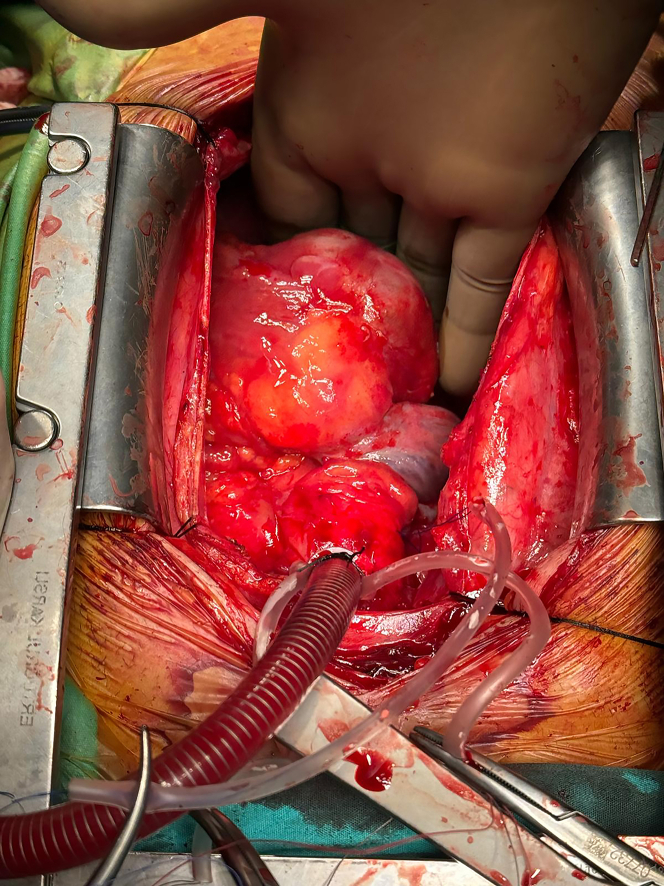
Mass Appearing on the Right Ventricle and Right Atrium

**Figure 6:**
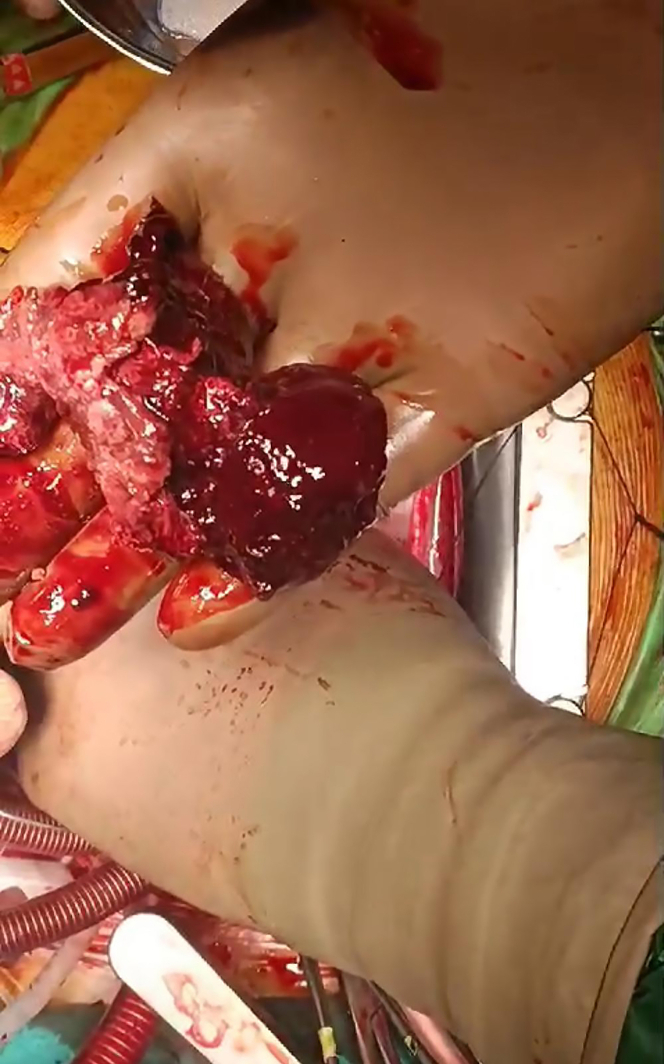
Excised Pseudoaneurysm Material

After the pericardium was opened and suspended, a 5 × 5 cm tumoral mass was observed in the RA, mostly over the RV (Figure 5). Bicaval cannulation of the aorta was performed considering the possibility of a mass image in the RA. The cava was rotated with a snare. The cross-clamp was placed, and cardiac arrest was achieved with antegrade cardioplegia. Because the mass was concentrated on the RV, it was thought that it was not in the RA and it was opened directly from the part close to the RV-RA junction. A pseudoaneurysm filled with organized and fresh thrombus was observed. Because of a previous myocardial infarction, a single saphenous bypass of the RCA was performed to the distal part of the tumoral mass. Part of the pseudoaneurysm sac and excisional material were sent for pathologic examination (Figure 6). A fistulized area was observed in the superior and middle parts of the sac, possibly in communication with the RCA. It was observed that the fistula originated from the site where the material from the saphenous vein was taken. The fistula openings were repaired using 6-0 pleated Prolene. After hemostasis was achieved, the sac was closed by suturing the ovary. Hot-shut cardioplegia was administered before cross-clamping. Afterward, the heart worked spontaneously, the patient came off the pump without any problem, and anatomical closure was achieved by controlling bleeding after decannulation.

## Outcome and Follow-Up

Pathology reports of the specimens obtained from the patient were compatible with pseudoaneurysm. The patient was discharged on dual antiplatelet therapy with acetylsalicylic acid and clopidogrel.

## Discussion

Coronary artery aneurysms are between 0.15% and 6%.^1^^,^^2^ Aneurysms are divided into true aneurysms, which are segments that are at least 50% wider than the proximal lumen, and pseudoaneurysms, in which the integrity of the vessel is compromised and there is a transition from a wall of 3 layers to a single-layer that expands outward.^3^^,^^4^

True coronary artery aneurysms can also be seen in conditions such as atherosclerosis, Behçet disease, Kawasaki disease, and endocarditis, whereas coronary artery pseudoaneurysms are usually seen iatrogenically after coronary stent implantation or coronary bypass graft.^1^^,^^5^, ^6^, ^7^ The absence of an etiologic cause in our patient made him an interesting case.

Coronary pseudoaneurysms may cause symptoms due to impaired flow or mass compression. The symptoms depend on the coronary artery where the pseudoaneurysm is located, the size of the pseudoaneurysm, and the structure it compresses, and include chest pain, shortness of breath, palpitations, and syncope.^8^^,^^9^ Our patient presented with chest pain and STEMI because of RCA-related flow disturbance.

Echocardiography, coronary CTA, cardiac magnetic resonance, and coronary angiography are used in the diagnosis of CAP.^8^^,^^10^ As coronary CTA and cardiac magnetic resonance are examinations that are performed by appointment and have special procedures in our hospital and our patient had STEMI and persistent chest pain and was hemodynamically hypotensive, pulmonary CTA, which can be performed as an emergency in our hospital, was performed after echocardiography to provide faster information and guidance. In addition, coronary angiography was performed within the time recommended by the guidelines due to STEMI.

In asymptomatic patients with pseudoaneurysm, antiplatelet therapy or anticoagulant therapy is recommended depending on the location and size of the pseudoaneurysm.^5^^,^^8^^,^^11^ Depending on the location and size of the pseudoaneurysm, coil embolization, vascular plug, and surgical treatment can be performed in the symptomatic patient.^5^^,^^8^^,^^10^ In our patient, surgical treatment was considered primarily because TTE showed a mass filling the RA, computed tomography showed a structure filling the RA and partially compressing the RV, vascularized masses could not be excluded, the diagnosis of pseudoaneurysm was not clear preoperatively but became clear after intraoperative findings and pathologic report, and the patient had severe chest pain and was hemodynamically hypotensive in the process.

## Conclusions

CAP is usually iatrogenic, but it should be kept in mind that it can also be non-iatrogenic and can be presented in a wide range of situations from asymptomatic to rupture resulting in death.

## Funding Support and Author Disclosures

The authors have reported that they have no relationships relevant to the contents of this paper to disclose.

**Take-Home Message**

• A coronary artery pseudoaneurysm is rare, but it can occur non-iatrogenically.

## Appendix

Video 1 Image of the Mass Filling the Right Atrium in the Patient's Transthoracic Echocardiogram

Video 2 The Patient's Right Coronary Artery Vessel on Coronary Angiography

## References

[1] Syed M., Lesch M. (1997). Coronary artery aneurysm: a review. Prog Cardiovasc Dis.

[2] Kapoor A., Batra A., Kumar S., Pandey S., Agarwal S.K., Sinha N. (2011). Coronary pseudoaneurysm in a non-polymer drug-eluting stent: a rare entity. Asian Cardiovasc Thorac Ann.

[3] Bhupali A., Joshi A., Patil S. (2015). Giant coronary artery pseudoaneurysm after drug-eluting stent implantation. Int J Clin Med.

[4] Caruso M., Evola S., Fattouch K. (2011). Chest pain due to late huge coronary pseudoaneurysm following stent implantation. Intern Med.

[5] Li D., Wu Q., Sun L. (2005). Surgical treatment of giant coronary artery aneurysm. J Thorac Cardiovasc Surg.

[6] Kato H., Ichinose E., Kawasaki T. (1986). Myocardial infarction in Kawasaki disease: clinical analysis of 195 cases. Pediatr Cardiol.

[7] Suzuki A., Kamiya T., Kuwahara N. (1986). Coronary artery lesions in Kawasaki disease: cardiac catheterization findings in 1100 cases. Pediatr Cardiol.

[8] Dalal P., Varma D., Chakravorty R., Sethi S., Bailey S., Prasad A. (2018). Transcatheter embolization of a giant coronary artery pseudoaneurysm. Cardiovasc Revasc Med.

[9] Crawley P.D., Mahlow W.J., Huntsinger D.R., Afiniwala S., Wortham D.C. (2014). Giant coronary artery aneurysms: review and update. Tex Heart Inst J.

[10] Daralammouri Y., Fuhrmann J., Kunze T. (2013). Giant right coronary artery aneurysm with a huge intramural thrombus. J Thorac Cardiovasc Surg.

[11] Valdis M., Gelinas J., Guo L. (2015). Invasive methicillin-resistant staphylococcus aureus endocarditis resulting in right ventricular pseudoaneurysm and fistulation to previous bypass graft. Ann Thorac Surg.

